# Feasibility study of prospective follow-up of neurodevelopmental outcomes in pregnancies identified as high stress at 20 weeks gestation in a disadvantaged community

**DOI:** 10.3389/frcha.2026.1996002

**Published:** 2026-10-28

**Authors:** Kristin M. Steiner, Noah J. Signaigo, Brianna V. Becher, Nick I. Ahmed, Jordan Booker, Candice King, Jean Goodman, Andrea Goldstein, Malori Chrisman Bidding, Nicole Takahashi, Kerri P. Nowell, David Q. Beversdorf

**Affiliations:** 1 School of Medicine, University of Missouri, Columbia, MO, United States; 2 Department of Psychological Sciences, University of Missouri, Columbia, MO, United States; 3 Department of Biological Science, University of Missouri, Columbia, MO, United States; 4 Department of Obstetrics, Gynecology, and Women's Health, School of Medicine, University of Missouri, Columbia, MO, United States; 5 Thompson Center for Autism and Neurodevelopment, Columbia, MO, United States; 6 Department of Health Psychology, University of Missouri, Columbia, MO, United States; 7 Interdisciplinary Neuroscience Program, University of Missouri, Columbia, MO, United States; 8 Department of Neurology, Radiology, and Psychological Science, William and Nancy Thompson Endowed Chair in Radiology, University of Missouri, Columbia, MO, United States

**Keywords:** African American, autism, disadvantaged groups, language development, prenatal stress

## Abstract

**Introduction:**

The impacts and mechanisms of environmental factors, such as prenatal stress, on neurodevelopment are poorly understood. Our lab previously characterized maternal miRNA changes related to prenatal stress exposure and its association with autism spectrum disorder (ASD) and subsequently examined miRNA from saliva samples associated with stress during pregnancy. Eighty-three pregnant Black women (ages 18–40) at the time of their 20-week ultrasound were assessed for stress markers by stress survey questionnaires. miRNA analysis was performed on the 10 highest- and 10 lowest-stress subjects. Thirty-four miRNA had significant differential expression, with 5 being upregulated and 29 downregulated in the high-stress group. Predicted targets revealed significant enrichment in neurodevelopmental pathways, including forebrain development, sensory system development, and neuronal growth regulation.

**Methods:**

In the present study, we assessed developmental outcomes of the children from a the top tertile of this cohort for stress exposure based on surveys assessing general developmental progress, language development, and autism spectrum disorder screenings.

**Results:**

Fifteen of this top tertile (27 individuals) had agreed to further contact and provided contact information, and eight of these individuals responded to the outcome queries. This study supported the feasibility of follow-up in this setting. Additionally, we found that a majority of the children from the high stress category pregnancies took longer to develop language milestones, showed behaviors of concern, and even reported a diagnosis of autism after follow-up.

**Discussion:**

This provides further support for the interrelationship between stress exposure during pregnancy and neurodevelopmental outcomes and the feasibility of follow-up after pregnancy for this type of study in disadvantaged communities.

## 1 Introduction

Autism Spectrum Disorder (ASD) is a neurodevelopmental condition characterized by impairments in social communication (e.g., difficulties with perspective-taking, interpersonal synchrony, and flexible language), and repetitive behavior (1). In recent years, the prevalence of autism diagnoses has been steadily increasing, particularly in minority populations. According to a 2014 study by the Autism and Developmental Disabilities Monitoring Network (ADDM), 1 in 59 8-year-old children residing in 11 predefined geographic sites in the United States were diagnosed with ASD. As of 2014, non-Hispanic White children were 20% more likely than Hispanic children and 10% more likely than non-Hispanic Black children to be identified as having ASD according to ADDM surveillance criteria (2). In 2018, ADDM reported a rise in ASD prevalence with 1 in 44 8-year-old children being identified with ASD. However, with this rise in diagnoses, researchers found no difference in prevalence between Hispanic, non-Hispanic Black, Asian/Pacific Islander, or non-Hispanic White children (3). In a recent 2023 report, prevalence of ASD per 1,000 children was significantly higher in Hispanic (31.6), non-Hispanic Black (29.3), and Asian/Pacific Islander (33.4) compared to non-Hispanic White (24.3) 8-year-old children for the first time. While there is an increased rate of diagnosis in Hispanic and non-Hispanic Black children, the consensus is that this is a result of improvement in access to care and diagnostic tools across racial and ethnic groups.

We use the term Black in ongoing focus for mothers' stressors and potential relevance to children's ASD diagnoses. We focus on how racially Black women are likely to encounter a distinct set of environmental stressors in society, given their physical appearance and the ways others identify them as Black and as female. These include the ways Black women may experience discrimination across romantic, workplace, and healthcare settings; racialized and gendered schemas that discourage Black women from seeking support in the face of distress; and a history of pervasive medical abuses that have eroded trust in preventive and primary healthcare (4). These are stressors that are not “exclusive” to Black women but do tend to be distinct from and can be qualitatively distinct from the experiences of women from other racial backgrounds and from Black men. Thus, while greater exposure to stressors can impact health for any population, we are focused on a group known to encounter disparate and extensive stressors in the United States with consensus downstream implications for both maternal and child health (e.g., maternal and infant mortality, risks of morbidity) (5).

Historically, ASD in Black people may be under-diagnosed compared to other groups. The 2023 ADDM study reported 50.8% of Black 8-year-old children with ASD were classified as having ID, whereas only 31.8% of non-Hispanic White children and 34.9% of Hispanic children were classified as having ID. This discrepancy is proposed to be due to the under-identification of Black populations with ASD without corresponding ID (6). If Black people with ASD and without ID are under-identified, the observed proportion of ASD cases with co-occurring ID (50.8%) may be falsely elevated. This means many Black people who potentially have undiagnosed ASD are not receiving the care that they need for successful management of this diagnosis. Relatedly, this marginalized population may be at a higher risk of developing ASD due to the increased number of stressors that they face (7). Understanding environmental stressors and how they potentially influence the development of ASD, specifically in Black individuals, will help to minimize the under diagnoses in this population and facilitate optimal treatment.

Genetic factors substantially influence susceptibility to ASD, as reflected by monozygotic twin concordance rates of 60%–92% (8). However, environmental factors—including prenatal psychosocial stress (e.g., job loss, death of a loved one, transitions into new school or work roles)—have become an increasingly important focus of ASD risk factor research (9). Furthermore, exposure to environmental stressors *in utero* can determine the postnatal health of a child and can potentially have a negative effect on neurodevelopment, including ASD (9, 10). Maternal environmental stressors and the corresponding stressor intensity may lead to epigenetic modifications that predispose the fetus to develop autistic tendencies or ASD (11). Our previous work identified a range of epigenetic changes associated with ASD-like behaviors in mouse offspring in association with prenatal stress, including increased global DNA methylation, which would contribute to decreased gene expression, and decreased miRNA and gene expression were also detected (12). In our subsequent work, we identified a miRNA profile preferentially observed in mothers of children with autism associated with prenatal stress, in contrast with mothers of children with autism not associated with prenatal stress (13). However, in that study, the miRNA samples were collected from the mothers when the child already had been diagnosed with ASD. To better understand the biological implications of the stress profile present during pregnancy, we subsequently assayed the miRNA profile in a mouse model during pregnancy, identifying the prenatal stress-related profile associated with ASD-like behavior in offspring (14). We then shifted focus to a still understudied population in this research space, considering the prenatal profiles and postnatal outcomes for Black mothers and children. To begin to understand this relationship in the clinical setting, we recruited pregnant Black women during their 20-week ultrasound and asked them to fill out a questionnaire quantifying stressors experienced during pregnancy. In this previous work, miRNA analysis was performed on the 10 participants who reported the highest-stress (high stress group) and 10 participants who reported the lowest-stress (low stress group). Thirty-four miRNA had significant differential expression with 5 being upregulated and 29 downregulated in the high-stress group. Predicted targets revealed significant enrichment in neurodevelopmental pathways, including forebrain development, sensory system development, and neuronal growth regulation (15).

In the present study, we followed up with these participants regarding their children via phone call to determine the feasibility of follow-up studies, regarding whether stressors present during pregnancy were associated with the development of autistic traits or ASD in the children, in order to determine response rate to help optimize design for future studies of this nature.

## 2 Materials and methods

### 2.1 Participants

The pool of participants was taken from 83 pregnant Black women at their 20 weeks ultrasound from July 2022 to January 2023. All participants were seen at the University of Missouri's Thompson Center for Autism & Neurodevelopmental and Maternal-Fetal Medicine clinics. To be sure that the extremes of maternal age was not a confounding variable, women outside of the age group of 18 to 40 years, were not considered in the cohort. Additionally, to minimize potential confounds from genetic risk, women who had a child previously diagnosed with ASD were also not considered in the cohort. All procedures were conducted in accordance with the approval of the Institutional Review Board at the University of Missouri (IRB #2090123; IRB#2132016) 14 April 2022 and 8 July 2026 respectively, with waiver of consent approved for the follow-up surveys.

### 2.2 Prenatal procedures-stress surveys

The Holmes–Rahe Life Stress Inventory (also known as the Social Readjustment Rating Scale–SRRS) was completed by each participant at their 20-week ultrasound visit (16). This self-reported survey quantifies the overall stress levels of the participants and calculates the number of reported stressful incidences during the pregnancy. Examples of stressful incidences included troubles with their in-laws, major changes in responsibilities at work, change in residence, and similar events. The participants were asked to name the specific stressors they experienced, the duration of the stressor, and if they were experiencing the event at the time of the ultrasound appointment. Each identified stressor was recorded if it corresponded to a significant stressful event on the SSRS list. Events were then scored in severity based on the SSRS severity score. The participants were ranked, based on the number of incidences and SSRS scores, as a high stress individual or a low stress individual, as utilized in our previous work (17, 18). A Holmes score of 190 or greater indicated that a person had experienced high stress during their pregnancy.

### 2.3 Postnatal procedures and materials

We made efforts to follow-up with participants who indicated permission in the original stress survey and who met criteria for high stress exposure. Of the 83 original individuals that were screened for stress markers at the 20-week ultrasound, researchers identified the top tertile of individuals for stress exposure (27 individuals) as revealed from the surveys. From this list, 15 had agreed to be contacted for future study and had left contact information. All of these individuals had a Holmes score of 190 or greater. The preferred method of follow-up was via telephone call. However, when continual telephone calls failed after three attempts, an email was sent to the cohort as a secondary attempt. Participants who responded to follow up were asked to complete surveys regarding the behavior and development of the child they were pregnant with at the time of recruitment. This survey included questions from the Ages & Stages Questionnaires, Third Edition (ASQ-3), Ages & Stages Questionnaires: Social-Emotional, First Edition (ASQ-SE), The Modified Checklist for Autism in Toddlers, Revised with Follow-Up (M-CHAT R/F), the Parent Rated Likert Scale, and general questions regarding language development.

Among the general questions, parents were asked the age in months at which their child developed single words, the age in months at which their child began to used two-word phrases, if they were keeping up with others in school and play (yes/no), and if there were any behavioral issues noticed (yes/no). Parents were also asked openly if they had any concerns for their child. Demographic questions, such as sex of the child, gestational age at birth, and treatment of any pre-existing or new developmental or medical conditions were also included. Age was calculated based on estimated due date and gestational age of the child at birth.

The ASQ-3 is a measure of development intended for children aged 12 to 66 months. The aim of this survey is to evaluate the child on each of the following criteria: communication, fine motor, gross motor, problem solving (i.e., a measure of early cognitive development) and personal-social (i.e., a measure of early social skills), with particular interest in problem solving and personal-social (19). Parents select “yes”, “sometimes”, or “not yet” for individual items. Scores below developmental norms indicate a possible delay. The ASQ-SE is administered and scored in a similar way to the ASQ-3 and evaluates development in the following domains: self-regulation, compliance, communication, adaptive functioning, autonomy, affect, and interaction with people (20). Self-regulation was a specific measure of interest, evaluated with the targeted questions, “Does your child calm down after getting upset”, “does your child enjoy interacting with others”, “Does your child have frequent or intense tantrums” and “Does your child struggle with changes in routine”. Parents selected “often”, “sometimes” or “rarely” to these questions, with a lower score indicating a concerning signal.

The Parent Rated Likert Scale evaluates the parent's perception of the child's behavior and attitude. Parents are asked to rate their children's peer interaction and behavioral regulation on a scale of 1 to 5, with less than or equal to 2 being a concerning signal (21). These questions give a subjective parental assessment of the child's development. The M-CHAT R/F is an autism screening tool for toddlers that can be used up to the age of 48 months (22). For this screening phone interview, we focused on questions regarding whether the child makes eye contact during social interaction, whether they respond when their name is called, whether they point to show that they are interested in something, whether they bring objects to share, and whether they imitate actions like clapping or waving. Parents selected “yes” or “no”. An answer “no” was a risk response for these children. A score of 2 total indicated medium risk and need for monitoring, and greater than or equal to 3 was high-risk. High risk children are recommended for follow up evaluation.

## 3 Results

Of the 15 individuals contacted in the high stress group, eight responded to the survey and provided data regarding their children (Table 1). Five parents reported that their children were experiencing delays in language or development. Of the eight parents, three reported delayed use of single words (i.e., ≥24 months). Furthermore, two of the parents reported that their children had delayed development of 2-word phrases (i.e., ≥36months). Subjectively, parents identified some behaviors of concern as well. Parent 3 indicated that their child did not speak much until about age two. Parents 5 and 8 indicated that their children often had “frequent or intense tantrums”. While it does not meet criteria for concern, it is notable that 2 additional individuals indicated these tantrums “sometimes”. Finally, parent 5 was diagnosed with ASD prior to follow up according to the mother's report. Parent 5 did report hypertension in the mother during pregnancy, preterm labor, and a Neonatal Intensive Care Unit (NICU) stay, and participants 4 and 6 reported diabetes and hypertension in the mother during pregnancy, which may have been confounding variables. Only two children had none of these concerns in parent report. There were no new developmental or medical conditions that were reported for which any of the children were being treated.

**Table 1:** Participant survey responses.

	Participant 1	Participant 2	Participant 3**	Participant 4	Participant 5***	Participant 6	Participant 7	Participant 8 **	Mean	SD
Age (months)	44	45	41	43	48	43	44	43		
Age (years)	3.5	3.5	3	3.5	4	3.5	3.5	3.5		
Gestational age at birth (weeks)	41	40	41	39	28.5	37	44	39		
Gender	female	male	female	female	male	female	male	female		
Instances of Stress	14	13	7	7	7	5	4	5	7.75	3.73
Holmes-Rahe Life Stress Score	555	349	232	226	225	221	197	192	274.63	123.35
Development of Single Words (months)	8	24*	12	10	30*	11	24*	9	16	8.57
Development of Two Word Phrases (months)	11	24	18	18	36*	30	36*	24	24.63	8.96
Keeping up with others in school and play?	yes	yes	yes	yes	no*	yes	yes	yes		
Any behavioral issues you have noticed?	no	no	no	no	no	no	no	no		
ASQ-3 score	50	50	50	50	50	50	50	50	50	0
ASQ-SE score	40	30	35	40	20*	35	35	25	32.5	7.07106781
Likert Scale-Peer Interaction	4	5	5	5	4	4	5	5	4.625	0.51754917
Likert Scale-Behavioral Regulation	4	3	3	5	3	5	4	5	4	0.9258201
M-CHAT R/F	0	0	0	0	0	0	0	0	0	0

Table 1 shows maternal survey responses regarding children born to high stress pregnancies of Black women. Stress levels are indicated by the Holmes-Rahe Life Stress Score. Demographics and scores regarding language development, developmental progress (ASQ-3 and ASQ-SE), parent rated behavior (Likert Scale), and ASD screening (M-CHAT) are indicated for each child. Asterisks indicate a concerning marker for ASD behaviors. (M-CHAT R/F = Modified Checklist for Autism in Toddlers, Revised with Follow-Up, ASQ-3 = Ages & Stages Questionnaires, Third Edition, ASQ SE = Ages & Stages Questionnaires: Social-Emotional, First Edition, SD = standard deviation, * = meeting criteria for concern, ** = parent reported developmental delays of concern, *** = parent reports child was diagnosed with ASD).

* Indicates meeting criteria for concern.

** Parent indicated developmental delays of concern.

*** Child was diagnosed with ASD at age 2.5.

## 4 Discussion

These findings built upon our previous study of 83 Black expectant mothers with varying levels of prenatal stress, as measured by the Holmes–Rahe Life Stress Inventory, where we observed a miRNA profile associated with prenatal stress exposure during pregnancy (15). Prior studies have used this measure to research the association between prenatal stress exposure and subsequent ASD diagnoses (17, 18). Maternal stressors at 20 weeks gestation were quantified, and among the participants whose stress scores was within the top 1/3, 15 were contacted. Of the 15, eight participants were successfully reached by telephone or email and were able to provide information about their children's behavioral and language development. Therefore, this supports the feasibility of follow-up in studies of this type in this population. Furthermore, while this was predominantly a feasibility study, mothers who experienced high stress during their pregnancy reported data that coincided with developmental concerns. Five of the children in this study had parent reported developmental delays, with three parents reported their child to have reached their single-word milestones later than expected. A similar trend emphasizes this point, with two children reportedly developing later than expected in the milestone of the use of two-word phrases. Aside from two children being reported as having frequent or intense tantrums, one individual was diagnosed with ASD by report of the parent.

Three children in the present study were reported as having delayed acquisition of either single-word or two-word milestones. According to the CDC, children with typical development generally reach their one-word milestone between 12 and 18 months, while their two-word phrases typically emerge between 18 and 30 months (23). Delayed social communication is a core feature of ASD (1). Therefore, while not necessarily diagnostic in isolation, language delay is often considered a marker of concern for the development of ASD. Additionally, language is an important developmental domain, and delay can have significant effects on various behavioral and social aspects. Early language is crucial in regulating emotional responses and mapping social environments as the child develops. However, it is important to note the caveat that there were still multiple stress exposed pregnancies where no developmental concerns were raised, and most did not have autism diagnosed.

Prior literature has identified a positive association between prenatal stress and ASD, and the findings in this study appear to agree with this despite the small feasibility sample. A higher incidence of reported prenatal stressors had been reported during weeks 21–32 of gestation among mothers of children with autism, with a peak at 25–28 weeks (18). Furthermore, a meta-analysis of 15 studies found prenatal maternal stress to be associated with increased odds of ASD in offspring (pooled OR 1.64, 95% CI 1.15–2.34) (24). However, studies with only Black mothers are lacking. Black American women are reported to have higher levels of pregnancy and birth complications when compared to other racial and ethnic minority groups (25). A potential reason for this is the disproportionate levels of stress these mothers may experience when compared to other groups (7).

This study reveals interesing results, but a larger dataset is necessary to have sufficient statistical power to address questions regarding this association. Although many children in this study who were born to mothers with high prenatal stress portrayed ASD-related behaviors, the data set was limited, with a sample designed for assessment of feasibility, and these developmental aspects were only parent reported. These findings should only be considered hypothesis-generating, and are only descriptive, not inferential. Many participants did not answer the phone or respond to emails. Future studies can utilize ongoing engagement efforts, community engagement, and incentives to increase response rates.

## 5 Limitations

While this study targeted a particularly high-risk population, non-Hispanic Black families, future studies would need to contrast what is found with this group with other groups to better understand the effects. Additionally, to determine the specificity of the effect of stress, rather than demographics, in the incidence of developmental issues, adequately powered follow-up studies would need to focus on participation from a low stress comparison group. Records limited our ability to incorporate that group into the present study. The developmental findings of the present study are only descriptive, not inferential. Without a low stress comparison group it is not possible to fully understand whether any developmental concerns reported are specifically related to prenatal stress. Furthermore, contrasting mild vs. severe stress could provide valuable information in future studies to better understand phenotypical implications, and tracking other stress markers such as maternal cortisol to further assess the impact of stress would be beneficial as well.

While measures were taken to minimize the effects of confounding variables, their influence cannot be completely avoided. For example, one parent reported preterm labor leading to physical developmental delays as well as a NICU stay. Further, two parents reported maternal diabetes and hypertension during pregnancy. It is possible that these variables exerted some influence on the data, although the extent of their impact cannot be determined with this small data set. Maternal hypertension during pregnancy has been associated with autism (26), maternal diabetes has also been associated with changes in neurodevelopmental outcomes (27), and past studies have found an association between preterm birth and autism (28), but recent studies suggest that other factors may be driving this (29). Children are also at increased risk of autism after a NICU stay, but a multitude of factors may contribute to this association (30). We also acknowledge that the data collected is subject to response and recall bias given the method of collection. Individuals may have misinterpreted survey questions or inaccurately recalled developmental milestones in their child's early life. It is also possible that families with children with emerging neurodevelopmental disorders were more likely to respond to our calls, potentially biasing our findings. Such inaccuracies would have affected the reported data. Future studies should aim to avoid such limitations by expanding the sample size and demographics as well as controlling for pregnancy related complications that could confound, and also incorporating direct assessment of the children.

While the original study examined the effects of stress on miRNA during pregnancy, the contrast reported in that study was the highest 10 stress-exposed individuals vs. 10 participants with no reported stress exposure (15). While the present study drew from the same set of participants, there was insufficient overlap between the stress-exposed individuals in the original study and the stress-exposed individuals in the present study, as only a few of the highest 10 stress-exposed individuals were among the 8 respondents in the present study, limiting our ability to integrate the miRNA data into the present findings.

## 6 Conclusions

This promising data shows the feasibility of longitudinal studies concerning the link between environmental factors, particularly maternal stress, on neurodevelopment in a unique high-risk population. However, this should only be considered exploratory due to the limitations including small sample and lack of a comparison group. The developmental findings can only be considered descriptive, not inferential. Regarding feasibility implications, though we speculate that a similar study with a larger sample would require over-recruitment by at approximately 100% for those agreeing to follow-up and providing contact information, and by at least 250% for initial engagement not assuming agreeing to follow-up or providing contact information. Nonetheless, in this small, high stress exposure sample, a surprising number of children with neurodevelopmental concerns were observed, but this can only be considered hypothesis-generating as feasibility is the primary aim herein. Examining miRNA and other biomarkers from salivary samples may be a highly scalable method to further evaluate this data due to its minimally intrusive nature. miRNA biomarkers could help evaluate the interaction between prenatal stress as well as environmental exposures on neurodevelopment. This is of particular importance given that disadvantaged populations are at an increased risk of interaction with environmental exposures, such as air pollution, which is also associated with an increased risk of development of autism (31–34). Future studies should explore the involvement of biomarkers, and expand across demographic groups, to better understand this topic.

## Data Availability

The raw data supporting the conclusions of this article will be made available by the authors, without undue reservation.
